# Apoptosis and Glial Marker Profiles in Hippocampus and Amygdala of Medically Intractable Temporal Lobe Epilepsy

**DOI:** 10.1111/jcmm.71179

**Published:** 2026-05-14

**Authors:** Fatemeh Alipour, Sayed Mostafa Modarres Mousavi, Farshid Nourbaksh, Masoud Ghadipasha, Jaber Gharehdaghi, Christoph Kellinghaus, Erwin‐Josef Speckmann, Walter Stummer, Ali Gorji, Maryam Khaleghi Ghadiri

**Affiliations:** ^1^ Shefa Neuroscience Research Center Khatam Alanbia Hospital Tehran Iran; ^2^ Legal Medicine Research Center Legal Medicine Organization Tehran Iran; ^3^ Department of Neurology Klinikum Osnabrück Osnabrück Germany; ^4^ Department of Physiology I University of Münster Münster Germany; ^5^ Department of Neurosurgery University of Münster Münster Germany; ^6^ Epilepsy Research Center University of Münster Münster Germany; ^7^ Neuroscience Research Center Mashhad University of Medical Sciences Mashhad Iran

**Keywords:** brain, cognition, intractable epilepsy, seizure, trauma

## Abstract

Medically intractable temporal lobe epilepsy is associated with potential neuronal and glial damage in key brain structures, including the hippocampus and amygdala. This study aimed to examine apoptosis, caspase expression, and glial activation markers in these regions, and to evaluate their associations with clinical variables. Hippocampal and amygdala tissues from 17 patients with medically intractable epilepsy and autopsy‐derived non‐epileptic controls were examined for cellular apoptosis (TUNEL), caspase‐3, ‐9, and ‐12, as well as GFAP (astrocytic marker) and IBA1 (microglial marker) expression using immunohistochemistry. Correlation analyses were performed to evaluate associations between these molecular markers and various clinical variables. TUNEL‐positive cells and caspase‐3 expression were significantly increased in both the hippocampus and amygdala of patients with medically intractable epilepsy compared to control subjects. Caspase‐9 and caspase‐12 were selectively elevated in the amygdala, while GFAP was upregulated in both regions, and IBA1 showed no significant differences. Protein expression patterns varied with age, sex, psychiatric comorbidities, seizure type and frequency, traumatic brain injury, and antiseizure drug treatment. Age at seizure onset, epilepsy duration, and total seizure numbers were associated with distinct molecular profiles. The epileptic hippocampal and amygdala tissues exhibit distinct, region‐specific changes in apoptotic and glial markers. These findings suggest that apoptosis and glial alterations may contribute to region‐specific pathological mechanisms underlying medically intractable temporal lobe epilepsy and may be influenced by clinical disease characteristics.

## 1 Introduction

Epilepsy is a chronic neurological disease characterized by a persistent susceptibility to generate unprovoked seizures, affecting about 65 million people globally [1]. Epileptogenesis involves a complex interplay of pathological processes, including aberrant neurogenesis, astrocytosis, microgliosis, synaptic and network remodelling, neuroinflammation, extracellular matrix alterations, and both acute and delayed post‐seizure neuronal death [2, 3, 4].

In temporal lobe epilepsy (TLE), dysfunctions across neurons, astrocytes, and microglia converge with chronic neuroinflammation to disrupt network homeostasis, thereby promoting both the initiation and long‐term progression of epileptogenic processes [5]. Hippocampal sclerosis, characterized by neuronal loss and reactive astrogliosis within the Cornu Ammonis, is a hallmark pathological feature of TLE [6, 7, 8]. Moreover, the amygdala, which plays a critical role in TLE, may also be involved either alongside or independently from hippocampal sclerosis [9, 10, 11]. Astrocytes are crucial for neuronal survival and axonal outgrowth in both the peripheral and central nervous systems, and their dysfunction significantly contributes to epileptogenesis [12, 13]. In TLE, astrogliosis, marked by astrocytic hypertrophy and increased GFAP expression, can drive network hyperexcitability by impairing astrocytic glutamine supply for GABA synthesis and by altering GABAergic signalling from inhibitory to excitatory [14]. Similarly, microglial activation modulates neuronal activity and mediates neuroinflammatory processes implicated in seizures, late‐onset neurodegenerative processes, and aberrant neurogenesis [15, 16].

Investigations in human TLE and kainate‐induced rodent models of epilepsy indicate that hippocampal atrophy following chronic seizures may result from glutamate‐mediated excitotoxicity and caspase‐dependent apoptosis [17, 18]. Consequently, modulation of caspase activity has emerged as a potential therapeutic strategy, as it may limit seizure‐induced apoptosis and neuroinflammation [19]. Given the central role of morphological and molecular alterations in the hippocampus and amygdala in the epileptogenic network of human TLE [20, 21], alterations in GFAP, IBA1, and caspase expression may indicate underlying inflammatory and apoptotic mechanisms involved in epileptogenesis. This study aimed to (i) characterize astrocytic and microglial alterations (GFAP, IBA1), apoptotic changes (TUNEL assay), and caspase expression (caspases‐3, ‐9, and ‐12) in the hippocampus and amygdala of individuals with TLE, and to systematically compare these findings with those observed in autopsy‐derived control tissue; and (ii) to explore potential associations between these biomarker changes and patients' demographic and clinical characteristics.

## 2 Materials and Methods

### 2.1 Tissue Collection Procedures

All experimental procedures were reviewed and received approval from the Ethics Committee of the Shefa Neuroscience Research Center, Tehran, Iran. Written informed consent was obtained from all patients prior to inclusion in the investigation. Despite undergoing suitable trials with a minimum of two appropriately selected antiseizure drugs, these patients remained intractable to the treatment [22]. Following presurgical assessment, all patients were advised to undergo temporal lobe resection to control their seizures. Human hippocampal and amygdala specimens were obtained from 17 patients with medically intractable TLE (mean age 32.3 ± 2.8 years) during surgical resections performed in Khatam Hospital, Tehran, Iran, during the period 2011–2018. Demographic and clinical information was collected for all enrolled patients, including sex, age, age at seizure onset, seizure frequency, estimated cumulative seizure burden, epilepsy duration, antiseizure drug (ASDs) regimen, surgical outcome, presence of comorbid psychiatric disorders, hemispheric dominance, major predisposing risk factors for epilepsy (e.g., traumatic brain injury and febrile seizures), and magnetic resonance imaging findings (Table 1). Epileptic samples, overlapping with those in our previous study [11], were analysed here using a distinct set of molecular markers and methods focused on cell injury, as well as glial and caspase expression.

**TABLE 1:** Clinical history of epileptic patients.

No.	Gender/age (year)	Age of seizure onset (year)	Disease duration (year)	Seizure frequency (number of seizures)	Estimated total number of seizures	Drugs	ILAE classification	Psychiatric disorders	Side of lesion	Dominant lobe correlated to lesion	Risk factor of epilepsy	MRI
1	Male/43	19	24	Weekly (1)	1248	LEV, OCBZ	1	−	Left	+	Febrile Seizure	HS
2	Female/27	11	16	Weekly (3)	2496	LEV, LTG, CBZ	1	−	Left	+	None	HS
3	Male/14	1.5	12.5	Weekly (1)	650	LEV, VPA, OCBZ, PRM	3	−	Left	+	Trauma	HS
4	Male/23	13	10	Daily (1)	3650	LEV, VPA, CBZ	6	−	Left	+	None	HS
5	Female/37	7	30	Daily (2)	21,900	CBZ, LTG, PRM	1	+	Left	+	Febrile Seizure	HS
6	Female/52	7	45	Monthly (1)	540	CBZ, LEV	1	+	Left	+	None	HS
7	Male/50	18	32	Daily (10)	116,800	VPA, CBZ, TPM, PHB, PHT, PRM	1	+	Left	+	None	HS/dysplasia
8	Male/48	28	20	Monthly (1)	240	LTG, LEV, PHT, CBZ	1	−	Left	+	Trauma	HS
9	Male/30	4	26	Daily (10)	94,900	CBZ, VPA, LEV, TPM	1	+	Right	+	Trauma	HS
10	Female/17	5	12	Monthly (10)	1440	CBZ, VPA	1	+	Left	+	None	HS/dysplasia
11	Female/24	1	23	Weekly (10)	11,960	LEV, LTG	1	+	Right	−	None	HS
12	Male/18	14	4	Daily (5)	7300	CBZ, TPM	1	−	Left	+	None	HS
13	Female/39	4	35	Monthly (3)	1260	LEV, CBZ, LTG	1	+	Right	−	None	HS
14	Male/28	8	20	Weekly (2)	2080	CBZ, VPA, TPM	1	−	Right	−	Febrile Seizure	HS
15	Male/35	32	3	Monthly (2)	72	VPA, CBZ	1	+	Right	−	None	HS
16	Female/30	18	12	Weekly (2)	1248	LEV, LTG, CBZ, TPM	1	+	Right	+	None	HS
17	Male/34	3	31	Weekly (2)	3224	CBZ	1	−	Left	+	Trauma	HS

Abbreviations: CBZ, Carbamazepine; LEV, Levetiracetam; LTG, Lamotrigine; MRI, magnetic resonance imaging; OCBZ, Oxcarbazepine; PHB, Phenobarbital; PHT, Phenytoin; PRM, Primidone; TPM, Topiramate; VPA, Valproate.

Control tissues were obtained from autopsies conducted through the body donation program administered by the Forensic Medicine Organization, Tehran, Iran. This control cohort (*n* = 13; mean age 44.5 ± 3.1 years) consisted of individuals with no known history of psychiatric and/or neurological disorders. The causes of death among control subjects were cardiac arrest (*n* = 5), cardiorespiratory failure (*n* = 3), respiratory infection (*n* = 2), abdominal trauma (*n* = 2), and multiple organ failure (*n* = 1). Postmortem intervals for autopsy controls ranged from 2 to 8 h.

### 2.2 Apoptosis Assay

Apoptotic cells were identified using the Terminal deoxynucleotidyl transferase–mediated dUTP nick‐end labelling (TUNEL) assay with the In Situ Cell Death Detection Kit (Roche, Germany). Histological slices were deparaffinized, washed with xylene, and rehydrated through a graded ethanol series. Following a wash in 10 mM Tris–HCl (pH 7.6), the sections were incubated in methanol containing 0.3% hydrogen peroxide for 10 min to quench endogenous peroxidase activity. The sections were then treated with proteinase K (Roche, Germany; 20 μg/mL in Tris buffer) at 37°C for 30 min and incubated in TUNEL reaction mixture consisting of label solution (450 μL) and enzyme solution (50 μL) at 37°C for 60 min and then in horse‐radish peroxidase solution (Santa Cruz, Germany) for 30 min. The colourimetric reaction was developed using 3,3′‐diaminobenzidine (0.5 μL DAB with 1.5 μL peroxide buffer) for 5–10 min, followed by counterstaining with haematoxylin. Negative control sections were processed in parallel without the TUNEL enzyme to confirm staining specificity. Hippocampus and amygdala were examined under a light microscope (BX51, Olympus, Japan) connected to a digital camera (BX51, Olympus, Japan).

### 2.3 Immunohistochemistry Assessment

Tissue sections were obtained from each specimen using a systematic random sampling method. Paraffin‐embedded sections were deparaffinized and rehydrated via serial administration of alcohols. The sections (5 μm) were boiled for 3–5 min in 0.01 M citrate buffer (pH 6.0) for antigen retrieval. Endogenous peroxidases were blocked using 0.3% hydrogen peroxide for 30 min, and the sections were incubated with 10% NGS at room temperature for 1 h. After that, the sections were incubated overnight at 4°C with the following primary antibodies: caspase‐3 (Santa Cruz, Germany, 1:10, sc‐7148), caspase‐9 (Santa Cruz, Germany, 1:10, sc‐7885), caspase‐12 (Santa Cruz, Germany, 1:10, sc‐21747), IBA1 (Fujifilm Wako, Japan, 1:200), and GFAP (Sigma, Germany, 1:200, G3893). Following PBS washes, the sections were treated with horse‐radish peroxidase‐conjugated goat anti‐mouse (1:500; Abcam, UK, ab6785) and anti‐rabbit (1:600; Abcam, UK, ab6721) IgG secondary antibodies for 1 h at room temperature. The sections were developed in 3,3′‐diaminobenzidine (DAB) solution (Dako, Denmark) for 5–10 min to visualize immunoreactivity. Finally, the sections were dehydrated through graded alcohols and cover‐slipped with mounting medium. The slices were analysed using the light microscope (Olympus BX51, Japan) equipped with a Canon EOS digital camera. The number of caspase‐3‐, caspase‐9‐, caspase‐12‐, GFAP‐, and IBA1‐positive cells was quantified with ImageJ software (NIH, USA). The number of TUNEL‐, caspase‐3‐, caspase‐9‐, and caspase‐12‐positive cells relative to total cells, along with the number of GFAP‐ and IBA1‐positive cells per field, was quantified using ImageJ software. A consistent threshold was applied across all images within each marker group to ensure standardized detection of immunopositive cells. All quantifications were performed in a blinded manner with respect to experimental group assignment.

### 2.4 Statistical Assessment

Data were analysed statistically using GraphPad Prism software (version 8). Group differences in protein expression between control and epileptic tissues were examined by unpaired *t*‐tests for normally distributed data or Mann–Whitney *U* tests for non‐normally distributed data. Comparisons of more than two groups were performed by one‐way analysis of variance (ANOVA) followed by Tukey's post hoc test for parametric data, or the Kruskal–Wallis test for nonparametric data. Data normality was evaluated using the Shapiro–Wilk test. Receiver operating characteristic (ROC) curve analyses were applied to evaluate the ability of protein expression levels to discriminate between experimental groups. For markers with statistically significant area under the curve (AUC) values, optimal cut‐off thresholds were identified using Youden's J statistic to maximize sensitivity and specificity. Associations between variables were examined using Pearson's correlation coefficient for normally distributed data or Spearman's rank correlation coefficient for non‐normally distributed data. Differences in correlation strengths between epileptic patients and controls were assessed using *z*‐tests based on Fisher's *z* transformation. Multiple regression analyses employing both enter and stepwise methods (exploratory) were performed to determine whether marker expression levels in positive cells predicted clinical variables, including estimated total number of seizures, age at seizure onset, and epilepsy duration. Patients were ranked according to clinical variables and stratified into high‐ and low‐value groups, and exploratory subgroup analyses were performed to compare clinical variables with marker expression. To control for false positives arising from multiple comparisons, *p* values within each experimental set were adjusted using the Benjamini–Hochberg false discovery rate (FDR) method. Where appropriate, effect sizes (correlation coefficients) were shown to quantify the magnitude of the effects. Statistical significance was defined as *p* < 0.05.

## 3 Results

**FIGURE 1:**
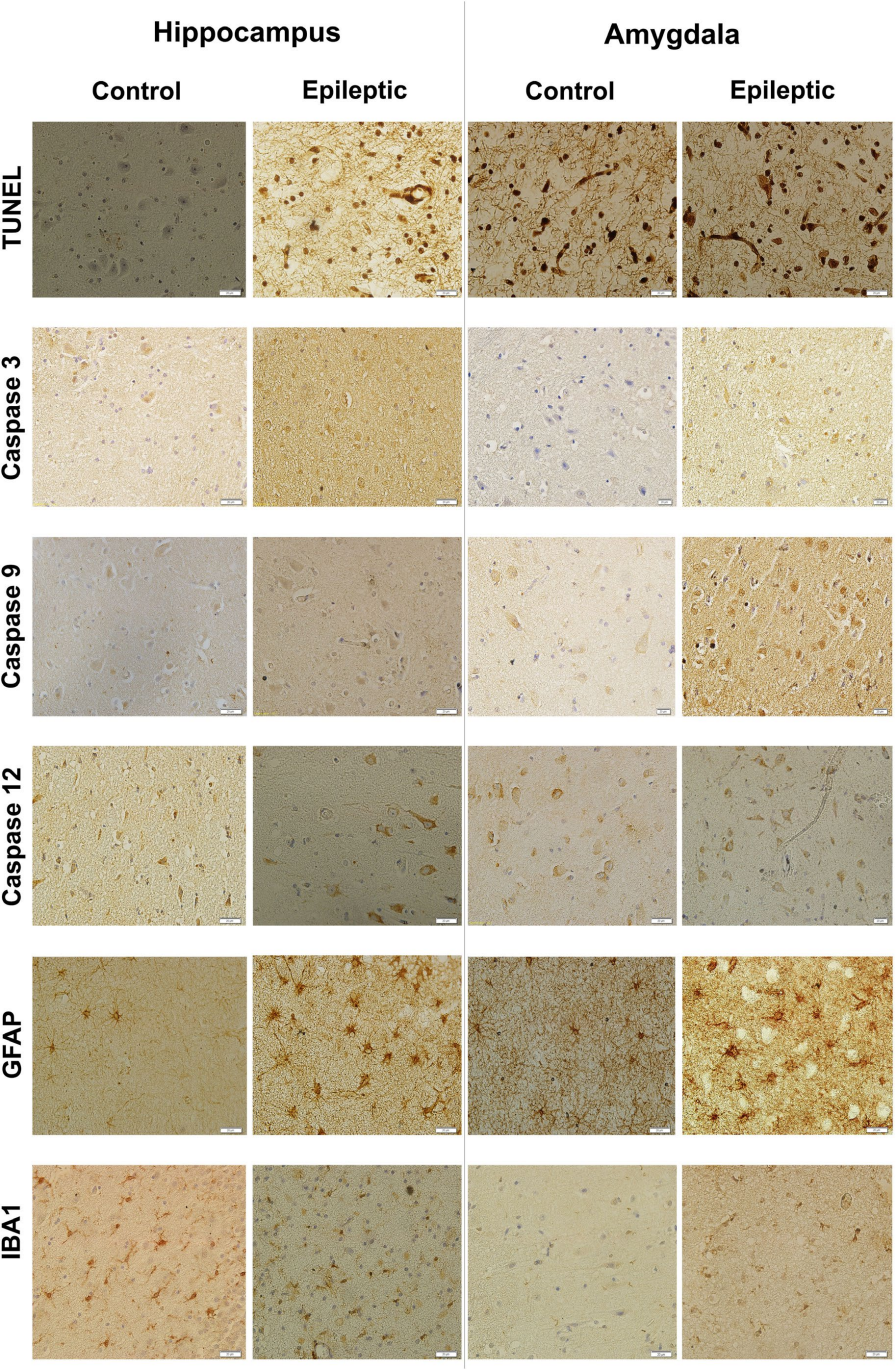
Immunoreactivity for the TUNEL assay and various protein markers (caspase‐3, caspase‐9, caspase‐12, GFAP, and IBA1) in hippocampal and amygdala tissues from control subjects and patients with epilepsy. Representative immunohistochemical images show TUNEL staining and immunoreactivity for apoptosis‐related markers (caspase‐3, caspase‐9, and caspase‐12) as well as glial activation markers (GFAP and IBA1) in the hippocampus and amygdala of patients with medically intractable epilepsy and autopsy‐derived non‐epileptic controls. Scale bars = 20 μm.

**FIGURE 2:**
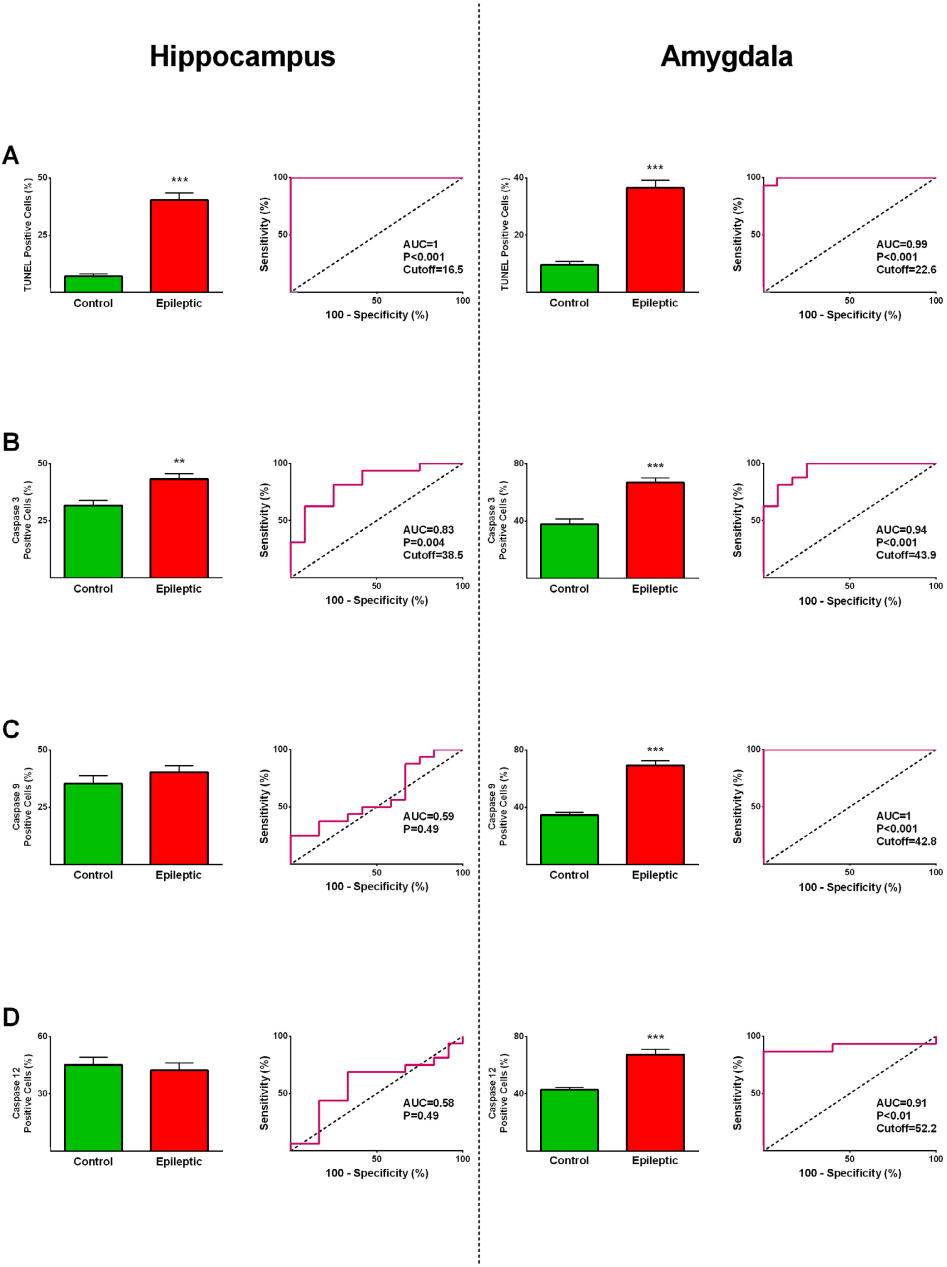
Apoptotic marker expression and ROC curve analysis in the hippocampus and amygdala of epilepsy patients versus autopsy‐derived non‐epileptic controls. Quantification of TUNEL‐ (A), caspase‐3‐ (B), caspase‐9‐ (C), and caspase‐12‐positive cells (D) revealed significant differences between epileptic and control tissues in the amygdala. In the hippocampus, the number of TUNEL‐ (A) and caspase‐3‐positive cells (B) was significantly different between the two groups. Data are shown as mean ± SEM. **, and *** denote *p* ≤ 0.01 and *p* ≤ 0.001, respectively.

Caspases‐3, ‐9, ‐12, and TUNEL expression in the amygdala and hippocampus of 17 epileptic individuals were compared with autopsy‐derived non‐epileptic controls. The amygdala and hippocampus of individuals with epilepsy exhibited significantly higher TUNEL (*p* ≤ 0.001) and caspase‐3 (*p* ≤ 0.01) expression than those of control subjects (Figures 1 and 2A,B). Furthermore, caspase‐9 and caspase‐12 expression values were significantly greater in the amygdala of epileptic patients compared with the control group (*p* ≤ 0.001, Figures 1 and 2C,D), whereas no significant differences were observed in the hippocampus (Figures 1 and 2C,D).

**FIGURE 3:**
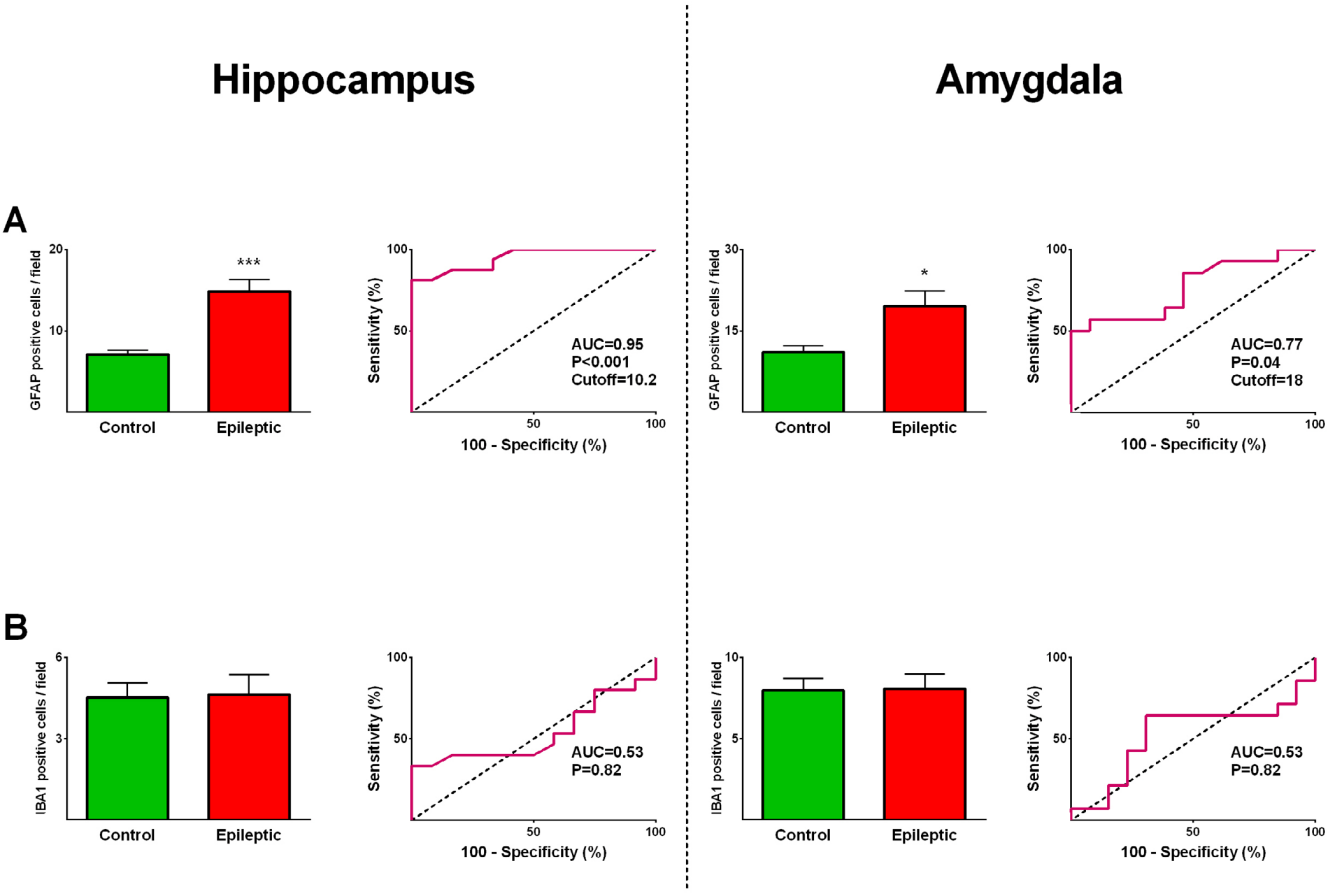
Glial marker expression and ROC curve analysis in the hippocampus and amygdala of patients with medically intractable epilepsy versus autopsy‐derived non‐epileptic controls. Quantification of GFAP‐positive cells revealed significant differences between epileptic and control tissues in both the hippocampus and amygdala (A). In contrast, the mean number of IBA1‐positive cells did not differ significantly between groups in both the hippocampus and amygdala (B). Data are shown as mean ± SEM. * and *** denote *p* ≤ 0.05 and *p* ≤ 0.001, respectively.

Furthermore, the expression levels of GFAP and IBA1 in the amygdala and hippocampus of epileptic and control specimens were evaluated. The expression values of GFAP in the epileptic hippocampus (*p* ≤ 0.001) and amygdala (*p* ≤ 0.05) were significantly greater than those of the control hippocampus and amygdala (Figures 1 and 3A). IBA1 expression in the amygdala and hippocampus was not significantly different between epileptic and control groups (Figures 1 and 3B). ROC curve analysis was performed to determine if the differentially expressed proteins could reliably discriminate between epileptic patients and non‐epileptic controls and to define the most appropriate cut‐off values. All markers that showed significant differences between the epileptic and control groups demonstrated statistically significant diagnostic performance, with AUC values ranging from 0.77 to 1.0 (Figures 2 and 3), indicating strong discriminatory potential.

### 3.1 Correlation Analysis of Caspases and TUNEL Immunoreactivity

**FIGURE 4:**
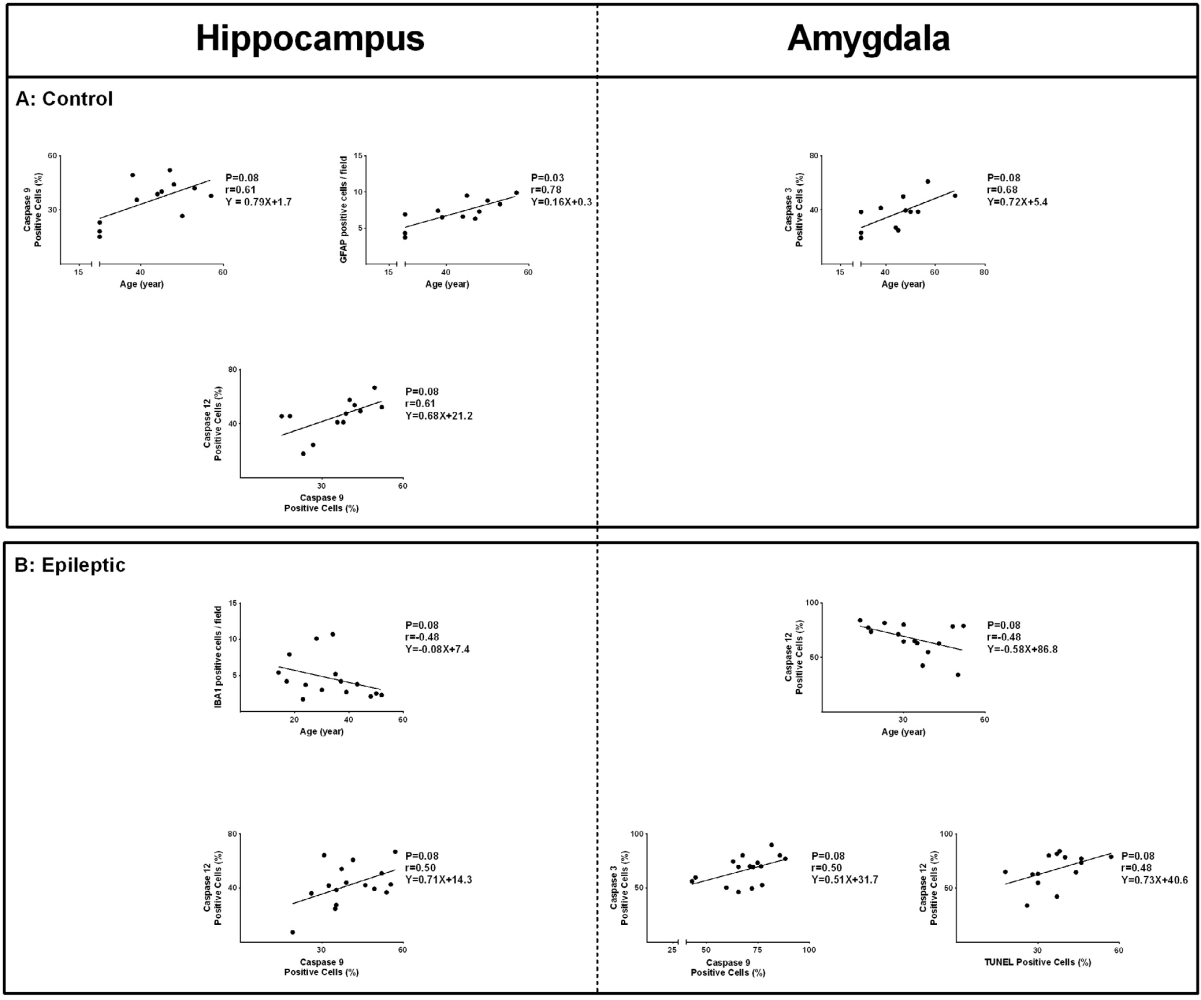
Correlation analyses of immunohistochemical markers with age and between various markers in hippocampal and amygdala tissues. Correlations between marker expression, demographic variables, and interrelationships among apoptotic markers were assessed in control and epileptic tissues. In controls, age was positively associated with hippocampal GFAP expression, with additional positive trends observed for hippocampal caspase‐9 and amygdala caspase‐3 expression with age, as well as between hippocampal caspase‐12 and caspase‐9 (A). In epileptic tissues, hippocampal IBA1 and amygdala caspase‐12 showed negative trends with age. Positive trends were observed between hippocampal caspase‐12 and caspase‐9, amygdala caspase‐3 and caspase‐9, and amygdala caspase‐12 and TUNEL‐positive cells (B).

Correlations between immunohistochemical marker expression and demographic variables, along with interrelationships among apoptotic markers in the amygdala and hippocampus, were assessed. In control subjects, increasing age was significantly associated with higher hippocampal GFAP expression (*r* = 0.78, *p* = 0.03; Figure 3A). Positive trends were observed for hippocampal caspase‐9 expression (*r* = 0.61, *p* = 0.08) and amygdala caspase‐3 immunoreactivity (*r* = 0.68, *p* = 0.08; Figure 3A) with age of controls. In addition, a positive trend between hippocampal caspase‐12 and caspase‐9 expression was observed in controls (*r* = 0.61, *p* = 0.08; Figure 4A).

In epileptic patients, hippocampal IBA1 expression showed a negative trend with age (*r* = −0.48, *p* = 0.08), and a similar inverse trend was observed between amygdala caspase‐12 expression and age (*r* = −0.48, *p* = 0.08; Figure 4B). Within this group, hippocampal caspase‐12 expression also demonstrated a positive trend with caspase‐9 expression (*r* = 0.5, *p* = 0.08; Figure 4B). In the epileptic amygdala, caspase‐3 expression exhibited a positive trend with caspase‐9 expression (*r* = 0.5, *p* = 0.08; Figure 4B), while caspase‐12 expression showed a positive trend with the number of TUNEL‐positive cells (*r* = 0.48, *p* = 0.08; Figure 4B).

### 3.2 Comparison of Correlation Coefficients

Fisher's *r*‐to‐*z* transformation was used to compare correlation coefficients between patients with epilepsy and controls. Analysis revealed a significant difference in the correlation between age and GFAP‐positive cells in the hippocampus between the two groups (*Z* = −2.36, *p* ≤ 0.02; Table 2). In the amygdala, a significant group difference was identified in the correlation between age and caspase‐3 expression (*Z* = −1.92, *p* ≤ 0.05; Table 2). Furthermore, correlations between caspase‐9 expression and IBA1‐positive cells (*Z* = 2.12, *p* ≤ 0.03; Table 2) differed significantly between epileptic patients and controls.

**TABLE 2:** Comparison of the correlation coefficients in the hippocampal and amygdala specimens.

Parameter A	Parameter B	Test statistic *z*	Probability *p* one‐tailed	Probability *p* two‐tailed
**Hippocampus**				
Age	GFAP	−2.36	0.009	0.02
**Amygdala**				
Age	Caspase 3	−1.92	0.03	0.05
Caspase 9	IBA1	2.12	0.02	0.03

### 3.3 Impact of Clinical Variables on Marker Profile in Epileptic Patients

**FIGURE 5:**
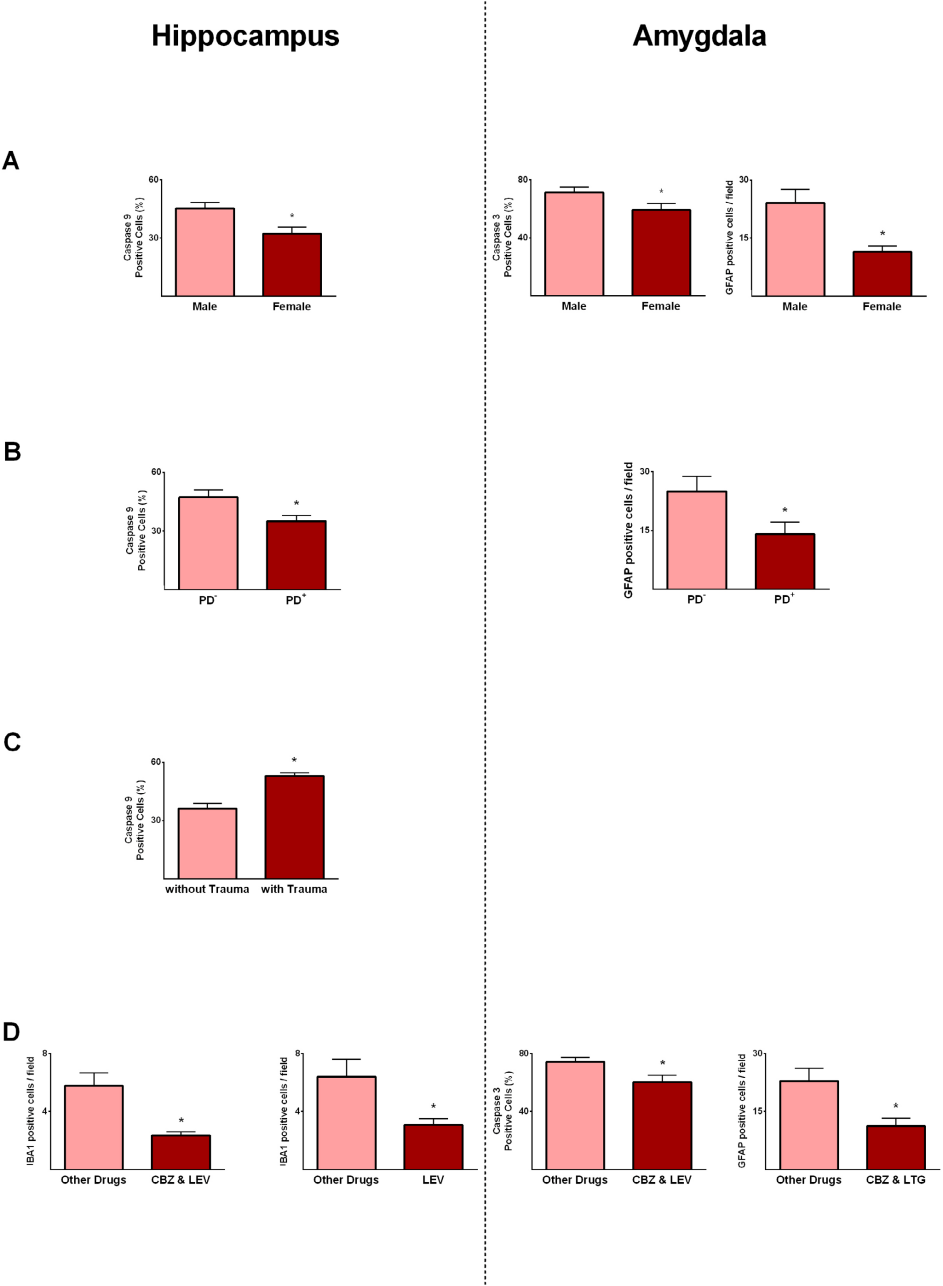
Expression of apoptotic and glial proteins in hippocampal and amygdala tissues of patients with medically intractable epilepsy in relation to demographic and clinical variables. Panels show comparisons based on sex (male vs. female, A), presence of psychiatric disorders (PD^+^) or absence of psychiatric disorders (PD^−^, B), history of traumatic brain injury (C), and effects of different antiseizure therapies (D). * indicates *p* ≤ 0.05. CBZ, Carbamazepine; LEV, Levetiracetam; LTG, Lamotrigine.

Female sex hormones play a critical role in epilepsy by modulating neuronal excitability, apoptotic signalling, and susceptibility to seizure‐induced neuronal and glial cell injury [23]. These hormonal effects underscore the importance of considering sex‐specific factors in epileptogenesis [24]. Among 17 patients with epilepsy, 10 patients were male, and 7 patients were female (Table 1). In epileptic patients, hippocampal caspase‐9 expression and amygdala caspase‐3 and GFAP levels were significantly lower in females than in males (*p* ≤ 0.05; Figure 5A). Neuroinflammatory processes in the limbic system are implicated in both seizure generation and psychiatric comorbidities of epilepsy, including anxiety and depression [25]. Among 17 epileptic patients, 9 had psychiatric comorbidities, primarily anxiety and/or depression (Table 1). To assess whether marker expression is associated with psychiatric disorders, we compared hippocampal and amygdala data between patients with and without comorbidities. Hippocampal caspase‐9 (*p* ≤ 0.05) and amygdala GFAP expression (*p* ≤ 0.05) were significantly reduced in epileptics with psychiatric disorders compared with those without (Figure 5B).

Traumatic brain injury (TBI) can drive epileptogenesis by triggering neuroinflammatory responses, including glial activation, cytokine release, and oxidative stress, which contribute to neuronal dysfunction and increased seizure susceptibility [26]. Among the seventeen patients, four had a history of moderate to severe TBI (Table 1). In these patients, hippocampal caspase‐9 levels were significantly elevated relative to those without a history of TBI (*p* ≤ 0.05; Figure 5C). ASDs can modulate apoptotic and necrotic signalling, potentially limiting neuronal and glial cell injury alongside their seizure‐controlling effects [27]. The ASDs most frequently administered to our patients were levetiracetam, lamotrigine, carbamazepine, and valproic acid (Table 1). Hippocampal IBA1 expression was significantly lower in patients receiving levetiracetam, either alone or in combination with carbamazepine, compared with those receiving other antiseizures (*p* ≤ 0.05; Figure 5D). In the amygdala, caspase‐3 levels were significantly decreased in patients on carbamazepine and levetiracetam (*p* ≤ 0.05; Figure 5D), while GFAP‐positive cells were significantly reduced in patients receiving carbamazepine and lamotrigine compared with other drugs (*p* ≤ 0.05; Figure 5D).

**FIGURE 6:**
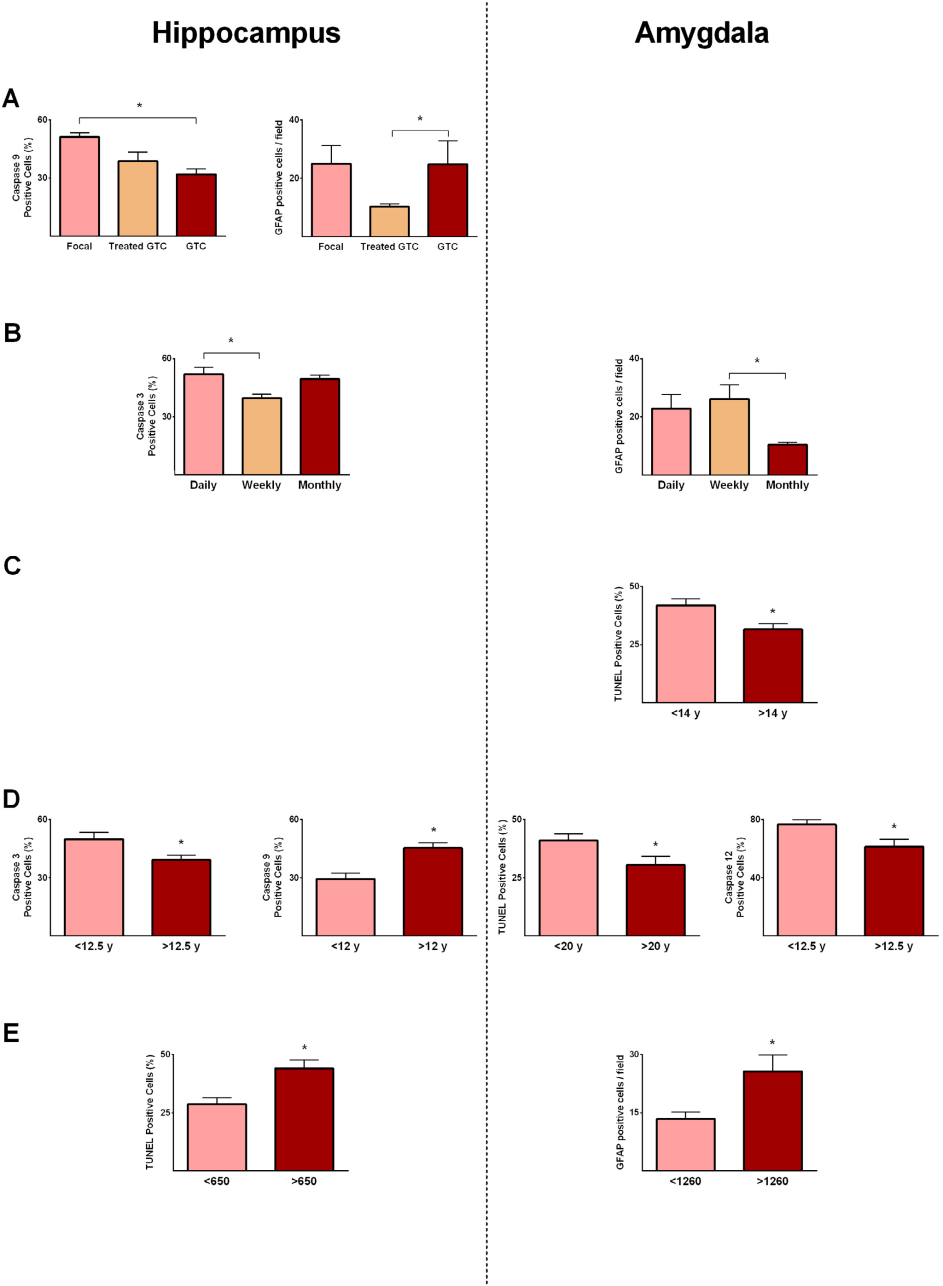
Expression of apoptotic and glial proteins in the hippocampus and amygdala of patients with medically intractable epilepsy in relation to clinical characteristics. Panels show comparisons based on seizure type (focal, treated, and untreated secondarily generalized tonic–clonic seizures (GTC)) (A), seizure frequency (daily, weekly, monthly, B), age at seizure onset (C), epilepsy duration (D), and estimated total number of seizures (E). * indicates *p* ≤ 0.05.

In our patients, seizures were categorized as focal, generalized tonic–clonic (GTC), or treated GTC, following ILAE definitions [28]. The effects of various types of seizures on the expression of different markers were assessed. Hippocampal caspase‐9 expression was significantly reduced in epileptics with GTC compared with those experiencing focal seizures (*p* ≤ 0.05; Figure 6A). Moreover, hippocampal GFAP values were significantly greater in GTC patients than in patients with treated GTC (*p* ≤ 0.05; Figure 6A). Furthermore, seizure occurrence was classified into daily, weekly, and monthly categories, and its effects on marker expression were assessed. Hippocampal caspase‐3 levels were significantly greater in those patients with daily seizures compared with patients experiencing seizures weekly (*p* ≤ 0.05; Figure 6B). In the amygdala, GFAP expression values were significantly greater in individuals with weekly seizures than in patients suffering from monthly seizures (*p* ≤ 0.05; Figure 6B).

In our cohort, the onset of seizures occurred between 1 and 32 years of age (mean 11.4 ± 2.2 years) (Table 1), and the mean age at the time of surgery was 32.3 ± 2.8 years (range 14–52 years). In the amygdala, analyses revealed that the number of TUNEL‐positive cells was significantly reduced in patients whose seizure onset occurred after 14 years of age compared with those whose onset occurred before 14 years (*p* ≤ 0.05; Figure 6C). The duration of epilepsy varied between 3 and 45 years (mean 20.9 ± 2.8 years) (Table 1). Hippocampal caspase‐3 levels were significantly lower in patients with epilepsy lasting > 12.5 years compared with those with < 12.5 years (*p* ≤ 0.05; Figure 6D), whereas caspase‐9 expression was higher in patients with epilepsy > 12 years than in those with shorter duration (*p* ≤ 0.05; Figure 6D). In the amygdala, TUNEL‐positive cells were significantly reduced in patients with epilepsy > 20 years relative to those with < 20 years (*p* ≤ 0.05; Figure 6D), and caspase‐12 expression was lower in patients with epilepsy duration > 12.5 years compared with individuals < 12.5 years (*p* ≤ 0.05; Figure 6D). Moreover, the total number of seizures was estimated based on seizure frequency and duration of epilepsy, ranging from 72 to 116,800 in our 17 patients (Table 1). Hippocampal TUNEL‐positive cells were significantly increased in individuals with > 650 estimated seizures compared with those with < 650 (*p* ≤ 0.05; Figure 6E). GFAP‐positive cells in the amygdala were significantly higher in subjects with > 1260 total seizures than in patients with fewer (*p* ≤ 0.05; Figure 6E).

### 3.4 Clinical Correlates of Epilepsy‐Associated Markers

We evaluated whether expression values of the investigated biomarkers can predict clinically relevant variables, including epilepsy duration, age at seizure onset, and estimated total seizure burden. Multiple linear regression analyses identified amygdala caspase‐12 expression as the strongest predictor of total seizure number among the assessed markers (Table 3).

**TABLE 3:** Assessment of relationships among all measured parameters in hippocampal and amygdala tissues using multiple regression.

	Models	Markers	Β‐coefficients	Variable *p* value	Model *p* value	Adjust *R* square
Hippocampus	Age of seizure onset					
	1	Tunel	−0.06	0.83	0.77	−0.17
		Caspase‐3	−0.32	0.37		
		Caspase‐9	−0.004	0.99		
		Caspase‐12	0.18	0.6		
	Epilepsy duration					
	1	Tunel	−0.1	0.74	0.99	−0.33
		Caspase‐3	−0.13	0.73		
		Caspase‐9	−0.15	0.72		
		Caspase‐12	0.1	0.78		
	Estimated total number of seizures					
	1	Tunel	**0.39**	0.19	0.67	−0.12
		Caspase‐3	0.18	0.6		
		Caspase‐9	0.18	0.65		
		Caspase‐12	0.05	0.88		
Amygdala	Age of seizure onset					
	1	Tunel	−0.17	0.67	0.87	−0.27
		Caspase‐3	0.36	0.38		
		Caspase 9	−0.22	0.59		
		Caspase‐12	0.08	0.83		
	Epilepsy duration					
	1	Tunel	0.24	0.42	0.17	0.25
		Caspase‐3	−0.56	0.09		
		Caspase‐9	0.51	0.13		
		Caspase‐12	−0.64	0.05		
	Estimated total number of seizures					
	1	Tunel	0.21	0.5	0.18	0.24
		Caspase‐3	0.14	0.66		
		Caspase‐9	−0.28	0.38		
		Caspase‐12	−0.67	**0.04**		
	Stepwise method					
	**1**	**Caspase‐12**	**−0.61**	**0.02**	**0.02**	**0.32**

*Note:* Bold values indicate that amygdala caspase‐12 expression was the strongest predictor of total seizure number among assessed markers.

## 4 Discussion

In the present study, caspase‐3 expression and the number of TUNEL‐positive cells were significantly elevated in the hippocampus and amygdala of patients with TLE compared with controls, consistent with experimental and clinical evidence demonstrating enhanced caspase‐3–mediated apoptosis in epileptic brain tissue in both animal models and human patients following prolonged seizures [29, 30, 31, 32, 33, 34]. In contrast, caspase‐9 and caspase‐12 were selectively upregulated in the amygdala, indicating region‐specific engagement of apoptotic signalling pathways. Positive, non‐significant correlations between caspase‐12 and caspase‐9 in the hippocampus, and between caspase‐9 and caspase‐3 in the amygdala, suggest a possible trend toward coordinated activation of the intrinsic apoptotic cascade. In rodents, caspase‐12 is activated during prolonged endoplasmic reticulum (ER) stress and can cleave procaspase‐9, which in turn activates procaspase‐3 to execute apoptosis [35, 36, 37, 38]. In humans, caspase‐12 is generally considered largely non‐functional under physiological conditions due to frameshift mutations and premature stop codons, with caspase‐4 proposed as the principal ER stress–associated caspase [39]. Accordingly, caspase‐12 does not appear to play a significant role in normal development or physiological cell death but may become relevant under specific pathological stress conditions [40]. Indeed, studies in human brain tissue from certain disorders, such as Creutzfeldt–Jakob disease, have suggested involvement of caspase‐12–related pathways in neuronal death, likely mediated through ER stress–associated apoptotic mechanisms [41]. The observed positive trend between caspase‐12 expression and increased TUNEL‐positive cells in the epileptic amygdala may suggest a potential role for ER stress–mediated apoptotic pathways in seizure‐induced cell loss; however, this interpretation remains speculative and requires further investigation.

Analysis of GFAP and IBA1 protein expression in epileptic and control tissues revealed that only GFAP was significantly upregulated in both the amygdala and hippocampus of epileptic patients. Epilepsy is associated with structural and physiological alterations in limbic regions, and injury‐induced biochemical, morphological, and functional changes in astrocytes (astrogliosis) are characterized by GFAP upregulation [14, 42]. Astrocytic dysfunction has been implicated in seizure generation, as direct astrocyte stimulation can induce epileptiform discharges [43]. In contrast, although studies in epileptic rodents report increased IBA1 in hippocampal microglia [16], under our experimental conditions, we identified no significant differences in IBA1 expression between epileptic and control patients in either the hippocampus or amygdala. IBA1 expression may not fully reflect microglial activation state, as it labels microglia across multiple phenotypes and cannot distinguish functional activation status [44]. Differences in assessment methods, such as cell counting versus morphological analysis, may also impact the detection of microglial activation and contribute to the lack of observed differences in IBA1 expression in our study. Furthermore, microglial activation in human epilepsy may be region‐specific and influenced by factors such as prior clinical interventions, seizure duration, and the extent of seizure‐induced cellular damage or death [16, 45, 46, 47]. A negative, non‐significant correlation between age and IBA1 expression was observed in the epileptic hippocampus, alongside evidence that levetiracetam treatment is associated with reduced IBA1 levels, suggesting that hippocampal microglial density and/or activation may decline with age and be modulated by antiseizure therapy. In epileptic cases, IBA1 labelling index negatively correlated with age [47], consistent with our findings of age‐related changes in IBA1 expression. Levetiracetam treatment was associated with a reduced proportion of IBA1‐positive microglia in the CA1, CA3, and dentate gyrus regions of streptozotocin‐treated rats, indicating attenuation of hippocampal microglial activation [48].

The lower GFAP expression in the amygdala of epileptic females compared to males in our study may reflect hormone–mediated modulatory effects on astrocytes. Astrocyte and glial cell densities in brain regions such as the hypothalamus, amygdala, and hippocampus exhibit sex‐dependent differences [8, 49]. Similarly, reduced caspase‐9 levels in the hippocampus and caspase‐3 expression in the amygdala of epileptic females relative to males may result from sex‐hormone–modulatory effects on apoptosis and caspase activity. Oestrogen opposes apoptosis by reducing activation of intrinsic and executioner caspases, including caspase‐9 and caspase‐3, across multiple cell types, indicating its role as an anti‐apoptotic regulator [50, 51].

In the current study, the possible effects of various seizure types, including focal, GTC, and treated GTC seizures, on protein expression profiles were also investigated. Our data showed that the patients with GTC exhibited lower levels of caspase‐9 in the hippocampus than the patients with focal seizures. It has been shown that up‐regulating caspase‐9 involvement in later stages of disease, when apoptosis is prevalent. Moreover, the more prolonged seizures characteristic of TLE that may progress to GTC seizures are much more likely to lead to cell loss. This may explain why the caspase‐9 in the patient's hippocampus with GTC seizures has been down‐regulated compared to focal seizures. The values of GFAP in the hippocampus of patients with GTC were higher than those of patients with treated GTC, indicating that the presence of reactive astrogliosis can be influenced by the type of seizure in epileptic patients. Accumulating evidence identifies gliosis as a fundamental component of epilepsy‐associated histopathological changes and suggests its involvement in the mechanisms underlying epileptogenesis across various forms of epilepsy [52].

Furthermore, our findings may indicate that patients with daily seizures exhibited higher caspase‐3 levels in the hippocampus compared with those experiencing weekly seizures. Moreover, the expression of GFAP in the amygdala was higher in patients with weekly seizures than in individuals with monthly seizures. Patients with a higher lifetime seizure burden (> 650 vs. < 650 seizures) showed increased TUNEL‐positive cells in the hippocampus and elevated GFAP‐positive cells in the amygdala (> 1260 vs. < 1260 seizures). These results may suggest that elevated seizure frequency correlates with enhanced activation of apoptotic pathways in the hippocampus and increased astrocytic activity in the amygdala. Previous clinical and experimental studies have shown that greater seizure frequency and severity are associated with increased expression of apoptotic markers, such as caspase‐3, in epileptic brain tissue, indicating enhanced activation of apoptotic pathways with longer epilepsy duration and more frequent seizures [53, 54]. Similarly, the density of activated microglia and elevated GFAP expression have been observed in epilepsy models and human epileptogenic tissue in relation to repetitive seizure activity, supporting the link between seizure burden and astrocytic activation [55, 56, 57].

Patients with seizure onset before age 14 exhibited higher TUNEL‐positive cell counts in the amygdala than those with later onset, while an epilepsy duration of more than 12 years was associated with increased caspase‐9 expression in the hippocampus. In contrast, individuals with epilepsy lasting > 20 years showed fewer TUNEL‐positive cells in the amygdala, along with reduced caspase‐3 in the hippocampus and decreased caspase‐12 in the amygdala, compared with shorter disease duration. These results may suggest that prolonged inflammatory conditions involving recurrent seizures modulate apoptotic and inflammatory pathways in a region‐ and duration‐dependent manner, with evidence that prolonged inflammation alters apoptosis‐related proteins and favours pro‐survival signalling, consistent with dysregulated apoptosis [58, 59]. Caspase‐3‐mediated cleavage of G protein‐activated inwardly rectifying potassium channels may lead to down‐regulation of their function and expression, potentially exacerbating hippocampal cell injury during prolonged epileptic seizures and contributing to progressive memory deficits and cognitive decline [60].

TLE with psychiatric comorbidities represents a condition in which differential neuroinflammatory interactions may drive region‐specific alterations in glial reactivity and structural changes in limbic circuits, contributing to pathological outcomes [61, 62]. Our data show that epileptic subjects with a history of psychiatric disorders exhibit lower GFAP expression in the amygdala and reduced caspase‐9 levels in the hippocampus compared with those without such comorbidities. Furthermore, we found that the hippocampus of epileptic patients with TBI showed greater levels of caspase‐9 compared with those without TBI. Elevated caspase‐9 expression in the temporal cortex of individuals with TLE, associated with seizure severity and recurrence [63], as well as increased caspase‐9 levels in the cerebrospinal fluid of patients following TBI [64], has been reported previously.

A potential limitation of the present study is the age difference between the surgical TLE cohort and the autopsy control group. As the expression of proteins such as GFAP and caspases may be influenced by age [65, 66], this represents a potential confounding factor. Due to the limited availability of suitable autopsy tissue, age matching was not feasible. Although the observed differences are consistent with disease‐related changes reported in the literature, the influence of age cannot be excluded and should be considered when interpreting the results. Moreover, the relatively small sample sizes in certain subgroups, including specific drug combinations and seizure types, and the use of multiple regression analyses with limited observations may reduce statistical power and increase the risk of overfitting. Therefore, these findings should be considered exploratory and interpreted with caution, as they require confirmation in larger, independent cohorts. A further limitation is that the postmortem interval of control tissues, although relatively short [67, 68], may still influence protein expression due to hypoxia‐related and early autolytic changes, and thus should be considered when interpreting group differences.

## 5 Conclusion

In sum, our findings suggest that epilepsy is characterized by region‐specific activation of apoptotic and neuroinflammatory pathways in the hippocampus and amygdala, marked by increased apoptosis and altered glial reactivity. These molecular signatures are further modulated by psychiatric comorbidities, traumatic brain injury, seizure type, age, and ASD exposure, suggesting their potential dynamic and heterogeneous nature. Importantly, the sensitivity of apoptotic and glial markers to clinical variables highlights their potential therapeutic significance.

## Author Contributions

**Christoph Kellinghaus:** methodology, writing – review and editing. **Farshid Nourbaksh:** methodology, formal analysis. **Jaber Gharehdaghi:** methodology. **Ali Gorji:** conceptualization, supervision, visualization, writing – original draft, writing – review and editing. **Maryam Khaleghi Ghadiri:** conceptualization, methodology, supervision, visualization, writing – review and editing. **Walter Stummer:** methodology, writing – review and editing. **Sayed Mostafa Modarres Mousavi:** methodology, investigation, formal analysis. **Erwin‐Josef Speckmann:** visualization, writing – review and editing. **Masoud Ghadipasha:** methodology. **Fatemeh Alipour:** methodology, investigation, formal analysis.

## Funding

The authors have nothing to report.

## Conflicts of Interest

The authors declare no conflicts of interest.

## Data Availability

The data supporting the findings of this study are available from the corresponding author upon reasonable request.
